# Hemorrhagic cystitis: A challenge to the urologist

**DOI:** 10.4103/0970-1591.65380

**Published:** 2010

**Authors:** R. Manikandan, Santosh Kumar, Lalgudi N. Dorairajan

**Affiliations:** Department of Urology, Jawaharlal Institute of Post Graduate Medical Education and Research, Puducherry - 605 006, India

**Keywords:** Hemorrhagic cystitis, immunosuppression, radiotherapy, chemotherapy, viral cystitis, intravesical therapy

## Abstract

Severe hemorrhagic cystitis often arises from anticancer chemotherapy or radiotherapy for pelvic malignancies. Infectious etiologies are less common causes except in immunocompromised hosts. These cases can be challenging problems for the urologist and a source of substantial morbidity and sometimes mortality for the patients. A variety of modalities of treatment have been described for the management of hemorrhagic cystitis but there is none that is uniformly effective. Some progress has been made in the understanding and management of viral hemorrhagic cystitis. This article reviews the common causes of severe hemorrhagic cystitis and the currently available management options.

## INTRODUCTION

Hemorrhagic cystitis is defined as a diffuse inflammatory condition of the urinary bladder due to an infectious or noninfectious etiology resulting in bleeding from the bladder mucosa. The most common cause is bacterial infection that usually responds promptly to treatment. But chronic and recurrent hemorrhagic cystitis often arises from anticancer chemotherapy or radiotherapy for the treatment of pelvic malignancies. Infectious etiologies are less common causes of chronic hemorrhagic cystitis except in immunocompromised hosts like bone marrow transplant patients. These cases can be challenging and frustrating problems for the urologist and a source of substantial morbidity and sometimes mortality for the patients. This article reviews the important causes of recurrent hemorrhagic cystitis, its pathophysiology and the currently available management options to treat this disabling condition.

## MATERIALS AND METHODS

We searched PubMed™ for articles published in the last five years using the words “hemorrhagic cystitis” and those published in the last 10 years using a combination of key words for specific therapies and hemorrhagic cystitis. Relevant articles, including review articles and clinical trials, were selected from these. In addition, standard textbooks were also reviewed. Important cross references were also selected for the review.

## ETIOLOGY OF HEMORRHAGIC CYSTITIS

### Drug induced cystitis

A wide variety of agents including chemotherapeutic drugs are implicated in the development of hemorrhagic cystitis [Table 1]. The most important among these are the oxazaphosphorine compounds such as cyclophosphamide and ifosfamide (synthetic analogues) that are used in many chemotherapeutic protocols for cancers like solid tumors and lymphomas. Cyclophosphamide is also used in certain immuno-inflammatory conditions such as Wegener’s granulomatosis and rheumatoid arthritis. The dose-limiting toxicity with these agents is usually urinary tract toxicity. Urinary tract symptoms include storage lower urinary tract symptoms such as frequency, urgency, nocturia and dysuria. Microscopic hematuria occurs in 7 to 53% and gross hematuria in 0.6 to 15.0%.[1] Gross hematuria can range from light pinkish urine to exsanguinating hemorrhage.

**Table 1:** Causes of hemorrhagic cystitis

Drugs	Ifosfamide
	Cyclophosphamide
	Busulphan
	Thiotepa
	Temozolomide
	9-nitrocamptothecin
	Pencillin and its derivatives like methicillin, carbenicillin, ticarcillin, piperacillin
	Danazol
	Tiaprofenic acid
	Allopurinol
	Methaqualone
	Methenamine mandelate
	Gentian violet
	Acetic acid
Environmental toxins	Aniline dyes
	Toluidine
	Chlorodimeform
	Ether
Radiation	-
Infections	Viral infections like adenovirus, BK polyoma virus, herpes virus, cytomegalovirus, JC virus
	Bacterial organisms like *Escherichia coli, Staphylococcus saprophyticus, Proteus mirabilis, Klebsiella*
	Parasitic disease like schistosomiasis and Echinococcosis
	Fungal species like *Candida albicans, Cryptococcus neoformans, Aspergillus fumigatus, Torulopsis glabrata*
Other systemic conditions	Amyloidosis
	Immunoinflammatory diseases like Systemic lupus erythematosis, Rheumatoid arthritis and Crohn’s disease
	Boon’s disease
	
	

Hepatic microsomal cells cause the breakdown of cyclophosphamide to hydroxycyclophosphamide which is then converted to aldophosphamide by target cells. They undergo further metabolism to phosphoramide mustard, the active antineoplastic metabolite, and acrolein, which has no significant antitumor activity but is toxic to the urothelium.[2] Similarly, ifosfamide is metabolized to iphosphoramide mustard and acrolein. The bladder being a reservoir for urine is most vulnerable due to the prolonged exposure of its urothelium to acrolein. Acrolein causes release of inflammatory mediators such as tumor necrosis factor-alpha, interleukin-1 beta and endogenous nitric oxide[3] causing bladder mucosal edema, vascular dilatation and increased capillary fragility resulting in hemorrhage. In chronic cases progressive fibrosis of the wall can result in a small fibrotic non-compliant bladder.[4,5] This dose dependent toxicity occurs in 2 to 40% of patients treated with cyclophosphamide. The onset of hematuria usually occurs within 48 hours of treatment.[6,7]

Hemorrhagic cystitis is managed by stopping the drug or reducing the drug dosage. An alternative drug like azathioprine may need to be substituted in some of these patients.[8] Hydration and forced diuresis are used to reduce the toxicity profile of these agents. Continuous bladder irrigation (CBI) is also helpful in these patients as it decreases the duration of exposure of the urothelium to acrolein thereby reducing the toxicity. The drug sodium 2-mercaptoethane sulfonate (mesna) has also been used to prevent hemorrhagic cystitis caused by ifosfamide and less commonly by cyclophosphamide. Mesna is a sulfhydryl compound that is administered intravenously and rapidly excreted by the urinary tract where the sulfhydryl group of mesna complexes with the terminal methyl group of acrolein forming a nontoxic thioether.[2] Mesna is best given intravenously and administered in three doses. A loading dose equivalent to 20% (w/w) of the ifosfamide dose is given 15 minutes before the drug, followed by two similar doses 4 and 8 hours later. The half-life of mesna is 35 minutes. The side effects include diarrhea, headaches and limb pain.

The role of mesna in preventing cyclophosphamide-induced hemorrhagic cystitis is controversial although doses as high as 60 to 120% are being employed. A randomized controlled study has shown that mesna and hyperhydration are equally effective in preventing hemorrhagic cystitis associated with cyclophosphamide,[9] the incidence of hematuria in the two arms being 33 and 20% respectively (*P* = 0.31). Similarly, Vose *et al*., performed a prospective randomized study comparing mesna (100% of cyclophosphamide dose) with CBI (200 ml per hour). There was no difference in the 18% incidence of hemorrhagic cystitis, but the CBI group had a higher incidence of urinary tract infections and bladder spasms.[10] In a nonrandomized controlled clinical study, Hadjibabaie *et al*., reported that CBI in addition to mesna, hydration, and alkalization was beneficial in the prevention of hemorrhagic cystitis after allogeneic hematopoietic cell transplantation.[11] Hemorrhagic cystitis occurred in 50% of patients in the no CBI group versus 32% in the CBI group (*P* = 0.11). In the CBI group there was significant reduction in the mean duration of hemorrhagic cystitis (10 vs. 18 days; *P* = 0.02), duration of hospitalization (30.2 vs. 39.6; *P* < 0.001) and in late-onset hemorrhagic cystitis (*P* = 0.001). In general, CBI was well tolerated.

A study comparing combination of hyperbaric oxygen with mesna showed a 93% urothelial protection compared to 33% in the non-treated group.[12] Other compounds such as amifostine, glutathione, N-acetylcysteine and L-2- oxothiazolidine-4-carboxylate (Procysteine) have also shown promising results in controlling hematuria.[13] Other combinations of mesna with dexamethasone or glucose-mannose binding plant lectins have also shown proven benefit in controlling hemorrhagic cystitis.[14,15]

Malignant lesions, predominantly transitional cell carcinoma, occur in 2.0 to 5.5% of patients receiving oral cyclophosphamide for nonmalignant disease.[1] Development of squamous cell carcinoma, adenocarcinoma and leiomyosarcoma has also been reported.[16] Mesna may reduce the risk of bladder cancer.[17]

Other systemic chemotherapeutic agents less commonly cause hemorrhagic cystitis. Busulphan, an alkyl sulfonate compound used in the treatment of chronic granulocytic leukemia, has been reported to cause hemorrhagic cystitis in about 16% of patients. Alkylating agents like thiotepa, temozolomide, and 9-nitrocamptothecin (a topoisomerase I inhibitor) have also been implicated to cause hemorrhagic cystitis.[18] Certain medications like penicillins and its synthetic derivatives like methicillin, carbenicillin, ticarcillin, piperacillin and penicillin G,[19] on rare occasions, cause hemorrhagic cystitis through an immunological mechanism. Symptoms can take two weeks to develop after the medication is started. Urinalysis frequently reveals sterile pyuria, hematuria and eosinophiluria.[20] Immunofluorescent staining is significant for immunoglobulin G and M deposition in the submucosa of the bladder, suggesting an immune mediated hypersensitivity reaction. Danazol, a semi synthetic anabolic steroid, has caused hemorrhagic cystitis in 19% of patients with hereditary angioedema. Hematuria occurs after a long interval of symptom-free period and is unrelated to dose.[21] Tiaprofenic acid, a non-steroidal anti-inflammatory agent, is reported to cause hemorrhagic cystitis. The symptoms can occur within days of starting the medication or years later. The etiology may be due to direct toxicity to the bladder urothelium or due to immune mediated hypersensitivity reaction.[22] The main treatment in these cases is stopping the drug and control of lower urinary tract storage symptoms and the hematuria in these patients resolves in a few days.

Certain topical agents can cause hemorrhagic cystitis through direct irritation of the bladder mucosa. Accidental intraurethral insertion of nonoxynol-9 has been implicated in hemorrhagic cystitis. This is due to the acidic nature of the suppository (pH 3.5 to 4.5). Immediate bladder irrigation should be initiated to reduce the symptoms. Other medications like oxybutynin, hydrocortisone, dimethyl sulfoxide and intravesical 50 cc of 1% lidocaine and 100 mg of hydrocortisone helps in alleviating symptoms.[23] Ether has been injected into the balloon ports of Foley catheters in an attempt to deflate clogged ports. The balloon invariably ruptures and causes ether cystitis resulting in severe hematuria. Long-term sequelae include decreased bladder capacity and lower urinary tract storage symptoms.[24]

Other medications that have been implicated in the development of hemorrhagic cystitis include allopurinol,[25] methaqualone,[26] methenamine mandelate,[27] gentian violet[28] and intravesical instillation of acetic acid.[29]

### Environmental toxins

Occupational exposure to chemicals such as aniline (constituent of dyes, marking pens and shoe polish) and toluidine (found in pesticides and shoe polish) are known to cause hemorrhagic cystitis besides predisposing to developing urothelial cancer. Ingestion, inhalation or direct skin contact of the pesticide chlorodimeform, commonly used on cotton plants and fruit trees, can cause hemorrhagic cystitis which is due to its metabolite 2-methylaniline, an aniline derivative. Usually the hematuria is self-limiting once exposure to the offending chemical agent is eliminated.[30]

### Radiation

Radiation cystitis is a late complication of radiotherapy for pelvic malignancies like prostate and cervix and occurs at least 90 days after the initiation of radiation treatment but may occur in a delayed manner even beyond 10 years.[31] About 15 to 20% of patients treated with external beam radiation develop bladder-related complications.[32] There appears to be no correlation between the development of early and late radiation injuries.

Histological features include microscopic progressive obliterative endarteritis that leads to mucosal ischemia. The ischemic bladder mucosa then ulcerates and bleeding occurs. Neovascularity occurs in the damaged areas which causes the characteristic vascular blush on cystoscopic evaluation. The newly formed vessels are more fragile and bleed with bladder distension, minor trauma or any mucosal irritation. Submucosal hemorrhages and frank hematuria occurs. Acute episodes usually wane within 12 to 18 months in most of these patients.[33]

In contrast to acute changes, late radiation injuries are irreversible and progressive. The time interval between the treatment and development of delayed symptoms is inversely proportional to the dose received.[34] The pathophysiology of late radiation damage includes cellular depletion, fibrosis and obliterative endarteritis.[35] The fibrosis decreases the bladder capacity and patients present with lower urinary tract storage symptoms such as urgency, frequency and dysuria.

Radiation induced hemorrhagic cystitis is very difficult to treat because of the ischemic nature of the disease. Since there are no well controlled trials available comparing the existing treatment options, firm guidelines cannot be made.[36] Attempts to reduce radiation induced hemorrhagic cystitis using various oral agents such as steroids, vitamin E, trypsin and orgotein have not met with success. Currently, accurately tailoring the irradiation field and limiting the radiation dose to the bladder are employed in reducing the incidence of hematuria.

Hyperbaric oxygen (HBO) therapy has been extensively investigated in the management of radiation induced injuries. Initially introduced in 1953 as a radiosensitizer in radiation oncology, HBO was later found to decrease the radiation effects on various organs including the bladder.[37] HBO involves the inhalation of 100% oxygen pressurized to 1.4 - 3.0 atm in sessions of 60-120 min. Under these conditions, alveolar, arterial and tissue oxygen levels are driven to supraphysiologic levels, thereby stimulating angiogenesis, fibroblast proliferation and collagen formation.[38] Bevers *et al*., have reported the only prospective study so far on the role of HBO. The patients underwent 20 sessions of 100% oxygen inhalation at three bars for 90 minutes. At three months follow-up, an overall response rate of 92.5% was demonstrated among 40 patients who were refractory to standard measures. With a mean follow-up of 23 months, the recurrence rate of severe hematuria was 12% per year.[39] In another study employing an HBO protocol of 2.36 atm of absolute pressure with 90 minutes of 100% oxygen breathing per treatment for a minimum of 40 sessions, Chong *et al*., have reported that early intervention, within six months of hematuria, resulted in a superior therapeutic response in complete (96%) or partial (66%) resolution of symptoms (*P* = 0.003).[40] HBO is generally well tolerated. Initially concerns were raised regarding the risk of tumor growth because of HBO mediated angiogenesis, immunosuppression and free radical toxicity. Following a review of all the available literature in 2003, Fieldmeier *et al*., concluded that HBO had no more than a neutral effect on tumor growth.[41] The greatest drawback at present is probably the cost of treatment which amounts to $ 10,000-15,000 per patient in the United States.[42]

Hyaluronic acid at a dose of 40 mg/ml solution for 30 minutes as a weekly intravesical instillation was found to decrease the incidence of bladder complications by 33% when used as a preventive measure for the radiation cystitis.[43] WF-10, the intravenous formulation of a novel wound healing agent, tetrachlorodecaoxygen, has demonstrated some benefit in patients with wound healing disorders, including radiation cystitis.[44] It is an immune modifier and promotes wound healing through the inhibition of chronic inflammatory process. Among cervical cancer patients with grade 2 or 3 radiation cystitis, WF-10 showed a complete response rate of 74-88%. Comparing this with standard hematuria measures, WF-10 demonstrated a lower rate of hematuria recurrence (47% vs. 77%, *P* = 0.01) and a longer time to recurrence (300 days vs. 100 days, *P* = 0.004).[45] At present, the role of WF-10 in the management of radiation cystitis remains investigational.

### Infection

Pediatric and immunocompromised patients are susceptible to develop viral hemorrhagic cystitis. The BK polyoma virus, adenovirus types 7, 11, 34 and 35, Cytomegalovirus, JC virus and herpes virus have been implicated.[46,47] Polyoma virus is highly prevalent in the pediatric population and is thought to remain dormant and asymptomatic in the kidney and other organs after the initial infection. When the immune system is compromised, as in persons undergoing chemotherapy or chemical immunosuppression after bone marrow, stem cell and solid organ transplantation, the virus gets reactivated leading to cystitis. Polyomavirus has been reported to cause hemorrhagic cystitis in 5.7% to 7.7% of bone marrow transplant recipients. Onset is usually within one to four months after transplantation. Early diagnosis and treatment of viral cystitis may prevent significant morbidity of hemorrhagic cystitis. The diagnosis is based on molecular techniques, and real-time polymerase chain reaction, which allows quantification of viral load, is often the method of choice.[48] Although no drug is yet licensed for use in polyoma virus infection, cidofovir is becoming the drug of choice in viral hemorrhagic cystitis in immunosupressed patients because it is active against the most common viral pathogens. Leflunomide has been shown to significantly reduce BK viral load in blood and urine in renal transplant patients with biopsy-proven BK nephropathy. Although its use in BK associated-hemorrhagic cystitis has not been reported leflunomide may also be a potential agent to treat BK-virus associated cystitis.[49] Ciprofloxacin may have a prophylactic role in preventing BK viral cystitis in bone marrow transplant patients.

The most common bacterial causes are *Escherichia coli, Staphylococcus saprophyticus, Proteus mirabilis* and *Klebsiella* species.[5] Fungal organisms associated with hemorrhagic cystitis include *Candida albicans, Cryptococcus neoformans, Aspergillus fumigatus* and *Torulopsis glabrata*. On cystoscopic examination, whitish pseudo membranes or pale plaques may be seen covering the urothelium in candidal infection. The hemorrhage usually resolves in these cases with the treatment of the underlying condition. *Schistosoma haematobium* lives in the perivesical venous plexuses in infected humans and the eggs produced by the parasite are excreted into the urine or feces. The ova present in the urine implant in the mucosa resulting in hyperplasia and dysplasia and predispose to development of squamous cell carcinoma of urinary bladder.[50] *Echinococcus granulosus* infections can cause calcified cysts which may infiltrate the bladder wall causing hematuria.

### Systemic diseases

Hemorrhagic cystitis can occur rarely in systemic diseases such as amyloidosis, rheumatoid arthritis and Crohn’s disease.[51] Hematuria can occur in prolonged high-altitude air travel (Boon disease).[52]

## GENERAL PRINCIPLES OF MANAGING HEMORRHAGIC CYSTITIS

A grading system for severity of hemorrhagic cystitis has been proposed by Droller *et al*., for hemorrhagic cystitis.[53] The main use of this grading system is the standardization of scientific studies on this subject.

0 – No symptoms of bladder irritability or hemorrhage
1 – Microscopic hematuria
2 – Macroscopic hematuria
3 – Macroscopic hematuria with small clots
4 – Massive macroscopic hematuria requiring instrumentation for clot evacuation and/or causing urinary obstruction.

In all patients of hemorrhagic cystitis a thorough evaluation should be done to determine the cause. If an etiology is not obvious, the patient should undergo hematuria workup including urine cytology, upper tract imaging and cystoscopy. The patient’s current medications must be reviewed and anticoagulants must be stopped. Patients on chemotherapy may have thrombocytopenia and other coagulation abnormalities which must be corrected. Laboratory evaluation includes hemoglobin, complete blood count, blood urea, serum creatinine, and coagulation profile and urine culture. Patient should be hemodynamically stabilized with intravenous fluids and blood transfusion. Antibiotics are given until sterile cultures are obtained. Symptomatic treatment with drugs like oxybutynin and analgesics helps in alleviating symptoms

## TREATMENT OF INTRACTABLE BLADDER HEMORRHAGE

As a first step a large bore three-way Foley urethral catheter is inserted to decompress the bladder and start saline irrigation. This simple maneuver may slow or stop the bleeding altogether. In some instance cystoscopic clot evacuation may be necessary. During cystoscopy, bladder should be carefully evaluated for the source of bleeding and biopsy of suspicious malignant lesions or fulguration of bleeding spots can be done at the same time. Patients not responding to clot evacuation, and those with diffuse bleeding, require supplemental therapeutic techniques with systemic or intravesical agents.

### Medical management

Conjugated estrogens have been employed for the treatment of viral and radiation induced hemorrhagic cystitis. Estrogens are believed to act by stabilization of the microvasculature. Some series have described oral and intravenous administration of conjugated estrogens with success rates ranging from 60% to 86%.[54] Sodium pentosan polysulfates have been proposed due to its uroprotective qualities that help reduce the inflammatory response of the urothelium.[55] This compound replaces the surface glycosaminoglycans that have been depleted by the inciting agents and thereby decrease the bacterial adherence. This reduces the risk of infection which is often a trigger factor for hematuria. The initial dosage is 100 mg orally thrice daily, which is gradually reduced to a maintenance dose of 100 mg daily. It requires one to eight weeks to reduce the degree of hematuria and no side effects have been reported. HBO has also been used to control hemorrhagic cystitis arising out of several causes especially radiation or cyclophosphamide induced hemorrhagic cystitis but most of these are single case reports or small retrospective case series. Recently a case of successful use of HBO in BKV-associated hemorrhagic cystitis refractory to cidofovir has been reported.[56]

### Instillation therapy

E-aminocaproic acid inhibits fibrinolysis by preventing the activation of plasminogen to plasmin. It can be given orally, parenterally or intravesically by continuous bladder irrigation. Administration involves a loading dose of 5 g followed by 1 g/h for 8 h until bleeding stops. The maximum recommended dosage in 24 h is 30 gm. The major disadvantage of this treatment is the formation of hard clots that are not easily flushed from the bladder. The patient should be clot-free before starting this drug and should be used in conjunction with continuous bladder irrigation. Singh *et al*. reported a response rate of 92% in 37 cases by this treatment.[57] Upper tract hemorrhage is a contraindication to its use since clot formation within the ureter can lead to obstruction and acute renal failure.

Alum (aluminum ammonium sulphate or aluminum potassium sulphate) irrigation acts as an astringent that causes protein precipitation, vasoconstriction and decreased capillary permeability without damaging normal urothelium.[58] It is commonly delivered as a 1% solution (50 g alum in 5 liter sterile water) via CBI at a rate of 250 ml/h. It leads to complete resolution of hematuria in 60%-100% of patients.[59] The median time to resolution of hematuria ranges from three to four days, but therapy may be required for as long as seven days. Systemic toxicity is low as the urothelial permeability to aluminum is minimal. However, aluminum toxicity due to its absorption has been reported such as microcyctic hypochromic anemia, osteomalacia, dementia, encephalopathy, metabolic acidosis and coagulopathy, particularly in renal failure patients and in children.[60,61]

Silver nitrate bladder instillations cause a chemical coagulation and eschar at the bleeding sites. The solution is used in a concentration range of 0.5% to 1% and is instilled for 10 to 20 minutes in duration. Reflux should be ruled out before instillation as renal failure has been reported due to precipitation and obstruction of upper tracts.[62] Prostaglandin E1, prostaglandin E2 and prostaglandin F2 alpha or its synthetic analogue carboprost tromethamine have been found to be useful in the management of cyclophosphamide induced hemorrhagic cystitis[63] as well as BK viruria-associated hemorrhagic cystitis developing in patients after allogeneic bone marrow transplantation.[64] They have a cytoprotective effect by regulating mucus production.[65] They are also postulated to cause smooth muscle contraction of the blood vessels in the mucosa and submucosa via membrane stabilization. They can also induce hemostasis by platelet aggregation.[66] Bladder spasms are the only known side effect reported in 78% of patients.[63,64] The recommended starting dosage is 0.8 to 1.0 mg/dl.[67] Miodosky *et al*., have reported successful treatment of post-hematopoietic stem cell transplantation hemorrhagic cystitis with intravesical sodium hyaluronate with five out of seven patients achieving complete responses.[68] However, in a recent analysis of published literature on the use of hyaluronic acid in the treatment of hemorrhagic cystitis and other bladder conditions, Iavazzo *et al*., concluded that this modality of treatment has only limited effectiveness.[69]

Formalin (40% formaldehyde) is the best known and most effective intravesical hemostatic agent. When administered intravesically, formalin rapidly fixes the bladder mucosa through a process involving protein cross-linking. This prevents further necrosis and blood loss.[70] In view of the potential for significant treatment related morbidity, formalin instillation is reserved for intractable hemorrhage refractory to conservative treatment. Cystogram must be done to rule out vesicoureteric reflux and bladder perforation. Formalin must be instilled under a spinal or general anesthesia since it is caustic to the sensory nerves of the bladder. Preliminary cystoscopy is performed for clot evacuation and fulguration of bleeding vessels. The entire perineum should be painted with petroleum jelly and, in women; the vagina is packed with petroleum jelly gauze to protect the exposed skin and mucosa from the caustic effect of formalin. Through an 18F Foley catheter, the bladder is filled to capacity with 1-2% formalin under gravity at a pressure kept below 15 cm of water. Treatment session is generally limited to 15 min. Formalin can be administered intravesically in concentrations ranging from 1-10%. Approximately 10-30% of patients may not respond to low dose of 1-2% and may require a second instillation using high dose formalin (4-10%).[71]

Fibrin glue has been used by Ouwenga *et al*. in a patient with persistent hematuria refractory to all the recommended measures.[72] After cystoscopy and clot evacuation, 5 cc aliquots of fibrin sealant were applied through a 7F open ended catheter via air distension to the entire surface of the bladder. Some degree of hematuria resolution occurred. A list of instillation therapies and the dosage regimen is given in Table 2.

**Table 2:** Instillation therapies used in treatment of hemorrhagic cystitis

Drug used	Treatment protocol	Efficacy (%)	Side effects
E-aminocaproic acid	Loading dose of 5 g followed by 1 g/h for 8 h till hematuria resolves. Maximum dose per day is 30 g	92	Hard clot formation
1% Alum	250 ml/h	60-100	Microcytic hypochromic anemia, osteomalacia, dementia, encephalopathy, metabolic acidosis and coagulopathy
Silver nitrate (0.5% to 1%)	10-20 min	70	Renal failure
Phenol	100% phenol with 30 ml glycine	Not clear	
Prostaglandins	0.8 to 1.0 mg/dl, 45 to 60 min contact time	75-90	Bladder spasms
Formalin	1 %-2% formalin, at 15 cm water pressure, gravity instillation for15 min	70-85	
Hyaluronic acid	40 mg/ml solution for 30 min once a week	33	

### Embolization

Super selective embolization has been described as a treatment option for hemorrhagic cystitis refractory to conventional treatments. McIvor *et al*., reported successful control of severe hematuria in 22 of 25% patients (92%).[73] Gluteal pain, secondary to occlusion of the superior gluteal artery, is the most common complication due to pelvic embolization. This complication is minimal nowadays with the advent of superselective embolization using microcatheters and newer embolization particles.

### Surgery

**Figure 1:**
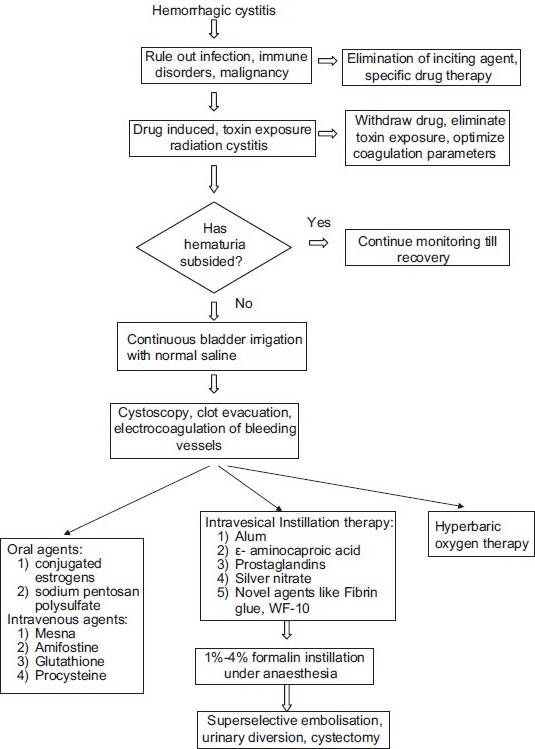
Management algorithm for hemorrhagic cystitis

Surgery is the last resort in patients of intractable massive hematuria. Various surgical procedures like urinary diversion including nephrostomy tube placement with occlusion of the ureteral orifices (balloons or glutaraldehyde cross-linked collagen),[74] cystostomy with ureteral catheters, ileal loop diversion, ureterosigmoidostomy and cutaneous ureterostomy, open packing of the bladder, ligation of hypogastric arteries and cystectomy and urinary diversion have been described. The goal of supravesical diversion is to decrease exposure of the hemorrhagic areas to urokinase to allow for hemostasis. Stillwell *et al*., reported the need for cystectomy for bleeding control in 5 of 100 (5%) patients with severe cyclophosphamide induced hemorrhagic cystitis.[7] An algorithm for the management of hemorrhagic cystitis is given in Figure 1.

## CONCLUSION

Although a variety of modalities of treatment have been described they are not uniformly effective in all conditions or patients. There is a paucity of well conducted scientific studies on the efficacy of these various treatments most of them being small case series. Some recent progress has been made in the understanding and management of viral hemorrhagic cystitis. There is a great need for better scientific studies to identify effective treatment modalities and preventive strategies to reduce the morbidity and mortality of hemorrhagic cystitis.
